# Spontaneous Twin Pregnancy in Uterus Bicornis Unicollis

**DOI:** 10.1155/2013/834952

**Published:** 2013-11-14

**Authors:** Arzu Doruk, Ilay Gozukara, Güneş Burkaş, Esin Bilik, Talat Umut Kutlu Dilek

**Affiliations:** ^1^Department of Obstetrics and Gynecology, Mersin University, School of Medicine, 33200 Mersin, Turkey; ^2^Department of Obstetrics and Gynecology, Kütahya Dumlupinar University, School of Medicine, 43050 Kütahya, Turkey

## Abstract

Abnormal fusion of the Müllerian ducts or failure of resorption of the septum causes varying degrees of congenital uterine malformation. They are often associated with reproductive problems such as miscarriage, premature labour, premature rupture of the membranes, or malpresentation. Twin gestation in a case of bicornuate uterus is extremely rare. A 37-year-old multiparous woman conceived a twin dichorionic diamniotic pregnancy spontaneously. Three-dimensional ultrasound revealed bicornuate uterus with one embryo in each cavity. Perinatal course was uneventful. At 35 weeks of pregnancy, spontaneous labour started and twin babies were delivered by bilateral low transvers caesarean section. Because of the rare occurrence of twin gestation in bicornuate uterus, there is no uniform guideline to manage these cases appropriately. Delivery by planned caesarean section could avoid the possible obstetric complications by dystocia.

## 1. Introduction 

Congenital abnormalities of female genital tract may involve fallopian tubes, uterus, cervix, and vagina. Uterine anomalies are the most commonly seen form Müllerian fusion anomalies. Uterine anomalies are associated with both normal or adverse reproductive outcomes [1]. Various occurance rates were reported by reproductive performance. In the fertile group, incidence was reported as 3-4%, 5–10% in women with recurrent early pregnancy loss, and up to 25% in women with late first or second trimester pregnancy loss or preterm delivery [2, 3]. Also these congenital anomalies are associated with late pregnancy adverse outcomes including preterm delivery and malpresentations [4]. Optimal way of pregnancy follow up is controversial in patients with Müllerian fusion anomalies. Spontaneous twin pregnancies in case of uterus bicornis unicollis were reported rarely. We reported an extremely rare occurance of spontaneous twin pregnancy in a women uterus bicornis unicollis.

## 2. Case

**Figure 1:**
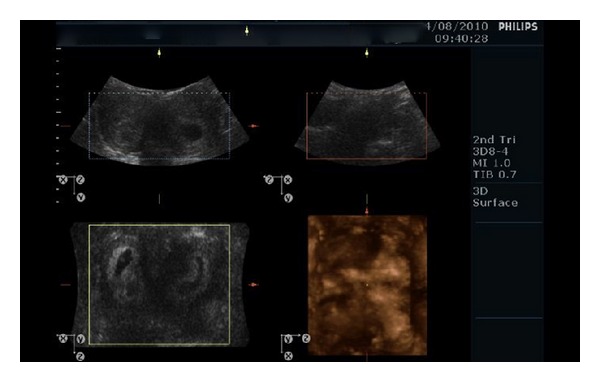
Two embryos were located in separate cavities of the bicornuate uterus.

**Figure 2:**
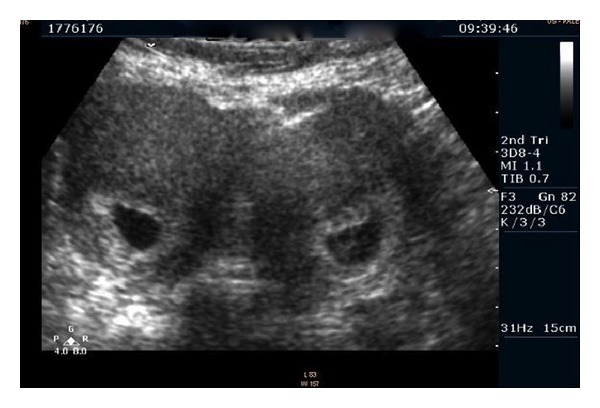
Two gestational sacs in coronal and sagittal view of 3D ultrasound.

**Figure 3:**
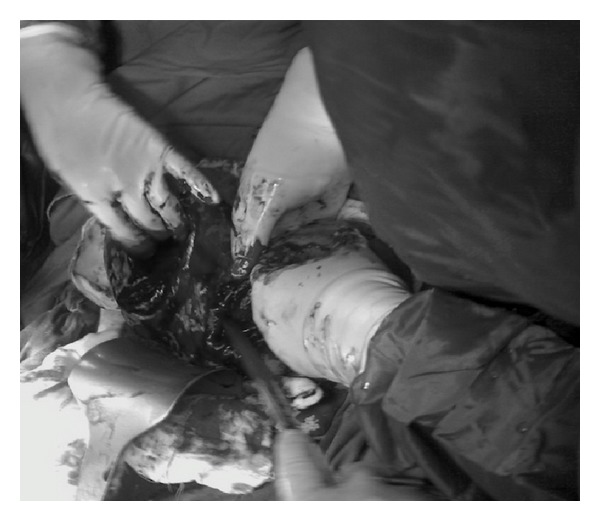
Two incisions were repaired separately.

A 37-year-old woman applied to hospital with twin pregnancy at 8th week of pregnancy. She was referred by suspicion of twin pregnancy that located in separate uterine cavities. During the gynecological exam, single cervix was detected. In the first ultrasound exam, a viable twin dichorionic pregnancy was seen. Both gestational sacs were located in separate uterine cavities. Crown-Rump Length (CRL) of embryos was 8,9 mm and 8,8 mm, respectively. Subsequent 3D ultrasound exam by Philips HD 11 SE which equipped 3D8-4 convex probe (Philips, Milwaukee, USA) revealed one twin in each uterine cavity (Figures 1 and 2) and that the uterus was bicornuate (Figures 1 and 2). Pregnancy outcome was uneventful. Down syndrome screening was performed by only nuchal translucency (NT) measurement and presence or absence of nasal bone. Down syndrome risk was calculated by NT for each twin separately. Anatomical survey by ultrasound was normal for each fetus. The patient was examined regularly in 2-3-week interval. Growth of both fetus was appropriate. At 35 weeks of pregnancy, she admitted by regular and painful uterine contractions. Cervical dilation was 2 cm and effacement was 50%. Both twins were in cephalic presentation. Presented part of fetus was over the pelvic inlet. She underwent caesarean-section due to previous C-section history. Both twins were delivered successfully by two separate lower segment transverse incisions. Birth weights were 2140 and 2270 g, respectively. Apgar scores were 7/9 and 8/9 at 1 and 5 minutes, respectively. Each incision was repaired simultaneously by two different operators with two rows of polyglactin sutures (Figure 3). There were no complications in the early and late postoperative periods. Total blood loss did not exceed 1000 mL (collected by surgical suction system and blood stained compress). She was discharged from the hospital 2 days after delivery with two healthy babies.

## 3. Discussion

Bicornuate uterus results from incomplete fusion of 2 uterine horns (Müllerian tubes) leading to varying degrees of separation between the cavities. Complete bicornuate uterus has two separate uterine cavities without any communication. Uterine anomalies have been associated with an increased incidence of spontaneous abortion, malpresentation, placental abruption, preterm delivery, intrauterine growth restriction, and the need for operative delivery [3, 5, 6]. The theories which were used to explain adverse pregnancy outcomes are diminished muscle mass, abnormal uterine blood flow, and cervical insufficiency [4].

We did not perform cervical cerclage to prevent second trimester pregnancy loss due to cervical insufficiency. She had two successful term deliveries. 

There are no guidelines about the followup of the pregnancy or selecting the mode of delivery, because the incidence is very low [7]. To date, there are only 12 reported cases of twin pregnancy in bicornuate uterus [7–11]. Mode of delivery could be abdominal or vaginal. However, dystocia, malpresentation, and possible risk of uterine rupture are major handicaps to avoid in the vaginal delivery [8].

We delivered twins by abdominal delivery. We performed first incision to deliver the first baby, and immediately a second incision was done to deliver the second one. Following the delivery of the fetuses and placentas, each incision was sutured simultaneously by two operators. 

Pregnancies of women with Müllerian anomalies have some potential obstetric complications. However, pregnancies within the bicornuate uteri have better obstetric outcome than other Müllerian fusion defects [3, 4]. The management of twin pregnancy in uterus bicornis should be individualized because of the possible risk and the rare occurance of these cases.
